# Effect of Urinary Protease Inhibitor (Ulinastatin) on Cardiopulmonary Bypass: A Meta-Analysis for China and Japan

**DOI:** 10.1371/journal.pone.0113973

**Published:** 2014-12-11

**Authors:** Yun Zhang, Zhi Zeng, Yu Cao, Xiaodong Du, Zhi Wan

**Affiliations:** Department of Emergency, West China Hospital, West China School of Medicine, Sichuan University, Chengdu, Sichuan, China; Harefield Hospital, United Kingdom

## Abstract

**Objectives:**

A meta-analysis was conducted to investigate the effects of ulinastatin treatment on adult patients undergoing cardiac surgery under cardiopulmonary bypass (CPB).

**Methods:**

Seven electronic databases were searched for reports of randomized, controlled trials conducted up to February 2014 in which patients undergoing cardiac surgery with CPB were administered ulinastatin in the perioperative period.

**Results:**

Fifty-two studies with 2025 patients were retained for analysis. The results showed that the ulinastatin can attenuate the plasma levels of pro-inflammatory cytokines and enhance the anti-inflammatory cytokine levels in patients undergoing cardiac surgery with CPB. Meanwhile, the ulinastatin had a significant beneficial effect on myocardial injury. The mean differences (MD) and 95% confidence intervals (95% CI) of biochemical markers were −63.54 (−79.36, −47.72) for lactate dehydrogenase, −224.99 (−304.83, −145.14) for creatine kinase, −8.75 (−14.23, −3.28) for creatine kinase-MB, and −0.14 (−0.20, −0.09] for troponin I (all P<0.01). However, neither hemodynamics nor cardiac function improved significantly, except that the MD and 95% CI of mean arterial pressure were 2.50 (0.19, 4.80) (P = 0.03). There were no statistically significant differences in the use of inotropes, postoperative bleeding, postoperative complications, the intensive care unit (ICU) stay, and the hospital stay; however, the frequency of auto resuscitation increased significantly (OR 1.98, 95%CI 1.19 to 3.30, P<0.01), the duration of intubation (MD −1.58, 95%CI −2.84 to −0.32, P<0.01) and the duration of mechanical ventilation (MD −3.29, 95%CI −4.41 to −2.17, P<0.01) shortened significantly in patients who were treated with ulinastatin.

**Conclusions:**

Ulinastatin can reduce the plasma levels of pro-inflammatory cytokines and elevate anti-inflammatory cytokine in patients from China and Japan undergoing cardiac surgery with CPB. Ulinastatin treatment may have protective effects on myocardial injury, and can increase the frequency of auto resuscitation, shorten the duration of intubation and mechanical ventilation.

## Introduction

A systemic inflammatory response (SIR) and multiple-organ ischemia/reperfusion injury often occur after open-heart surgery carried out under cardiopulmonary bypass (CPB) [1]. The activation of the complement cascade and fibrinolytic system by multiple physical, chemical, and biological stimuli in CPB, along with neutrophil activation and the generation of a sequential inflammatory cascade, contribute to a series of postoperative complications [2], [3], such as post-surgical bleeding, acute lung injury, acute respiratory distress syndrome, cardiac insufficiency, and acute renal injury [4], [5]. This results in prolongation of mechanical ventilation and ICU or hospital stay, and increases costs.

Ulinastatin is a broad-spectrum hydrolase inhibitor purified from the urine of healthy men. As a serine protease, ulinastatin is able to inhibit various inflammatory proteases, including trypsin, chymotrypsin, and neutrophil elastase and plasmin. Thus, it is considered an effective anti-inflammatory molecule, and it has been widely used clinically in China, Korea, and Japan to treat pancreatitis, rheumatoid arthritis, sepsis, and other inflammatory diseases [6], [7]. In recent years, ulinastatin has been used to prevent postoperative complications and post-pump organ injury in patients undergoing cardiac surgery with CPB [4], [8], [9]. Xu and colleagues found that high-dose ulinastatin can reduce pulmonary injury, improve pulmonary function after CPB, and shorten the duration of intubation and intensive care unit (ICU) stay [8]. However, Park and colleagues observed no significant effect of ulinastatin on major organ dysfunction, SIR, or other postoperative complications [9]. Since the effect of ulinastatin on patients' responses to CPB and the associated potential challenges and complications are not yet clear, we conducted a systematic review and meta-analysis to assess studies that used ulinastatin in order to evaluate its effect in patients undergoing open-heart surgery with CPB.

## Methods

### Search strategy

The electronic databases PubMed, EMBASE (using OVID), ISI, ACS, Cochrane Controlled Trials Registry, China National Knowledge infrastructure, and Wanfang Data were searched from their inception up to February 2014. The keywords and terms used were (“ulinastatin” OR “protease inhibitor” OR “UTI” OR “hydrolase inhibitors”) AND (“cardiopulmonary bypass” OR “cardiac surgery” OR “open heart surgery”). Papers in the English or Chinese language were included. All articles were imported into EndNote ×6 software to delete duplicates.

### Study selection criteria

The inclusion criteria for the primary studies were as follows: (1) randomized, placebo-controlled clinical trials; (2) adults undergoing open-heart surgery employing CPB; (3) ulinastatin was used perioperatively; (4) CPB time or aortic cross-clamping time were reported; (5) outcomes of interest included, ie, the inflammatory markers (TNF-α, IL-6, IL-8, and IL-10), the myocardial biomarkers: lactate dehydrogenase (LDH), creatine kinase (CK), creatine kinase-MB (CK-MB) and troponin I (TnI), the hemodynamic parameters heart rate (HR), mean arterial pressure (MAP), mean pulmonary arterial pressure (MPAP) and central venous pressure (CVP), cardiac index and left ventricular ejection fraction (LVEF) as markers of cardiac function, the numbers of patients needing inotropes and auto resuscitation, the postoperative bleeding, the postoperative complications, the duration of mechanical ventilation, the intubation time, and the lengths of ICU stay and hospital stay.

The exclusion criteria for the primary studies were as follows: (1) review, abstract, or case report; (2) animal or cell study; (3) not open-heart surgery; (4) pediatric cardiac surgery; (5) total number of patients <20, and (6) published in any language other than English or Chinese.

Study selection and data extraction were performed by two independent reviewers (YZ and ZW). Disagreements were resolved through consensus.

### Data extraction

A data collection form was designed before the data were extracted. The articles finally included were assessed independently by two reviewers (YZ and ZW). The extracted data included: (1) first author and year of publication; (2) total number of patients, number of patients in the ulinastatin and control groups, gender, age, body weight, and body surface area; (3) CPB time, and aortic cross-clamping time in both groups; (4) data regarding outcomes of interest, as described above, in the ulinastatin and control groups. When the results of the trial were reported as median and quartile, the Stela Pudar-Hozo method was used to estimate the mean and standard deviation [10].

### Assessment of study quality

Two reviewers (YZ and ZW) independently assessed the risk of bias, using the tool described in the Cochrane Handbook for Systematic Reviews of Interventions [11]. The risk of bias was rated as low, high, or unclear with regard to: (1) sequence generation; (2) allocation concealment; (3) blinding; and (4) completeness of outcome data.

### Statistical methods

Review Manager Software (version 5.02 for Windows; The Cochrane Collaboration, 2009) was used to perform the meta-analysis, and STATA 12.0 (Stata Corp, College station, TX) was used to evaluate the publication bias (Egger's test and Begg's test). In accordance with the Review Manager Handbook, odds ratio (OR) or mean difference (MD), and their 95% confidence interval (CI) were used to estimate the effective value. Statistical heterogeneity was examined by Cochrane Q-test (significant at P<0.1) and the I^2^ value. For comparisons with P<0.1 or I^2^<50%, a fixed-effect model was used; otherwise, a random-effect model was adopted. If necessary, a sensitivity analysis was also performed to evaluate the influences of individual studies on the final effect. All P-values were two-sided, and P<0.05 was considered significant.

## Results

### Search results and characteristics

**Figure 1:**
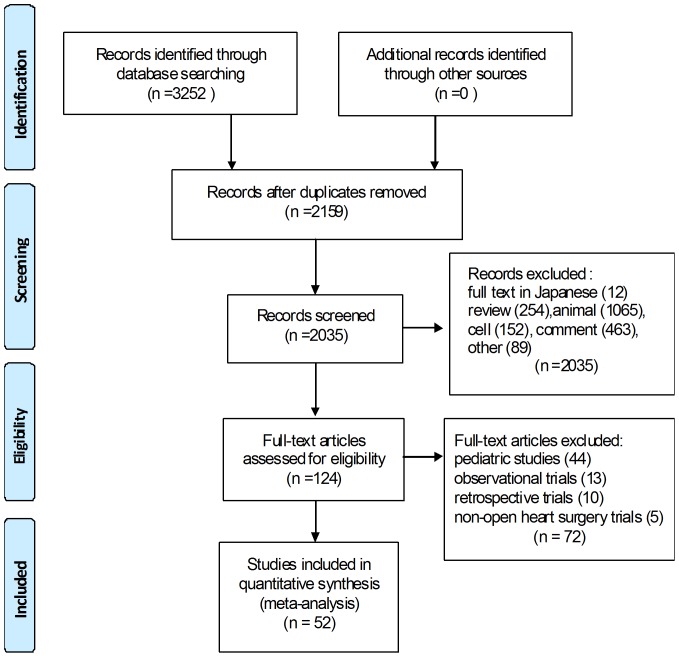
Flow chart showing how eligible studies were identified.

Of the initial 3252 records identified, 2159 remained after duplicates were removed. Two thousand thirty-five articles were excluded because they were not clinical trials (in Japanese, review, animal studies, cell studies, comment, etc.). Of the 124 articles reviewed at the full-text level, 72, including 44 pediatric studies, 13 observational studies, 10 retrospective studies, and 5 non-open heart surgery studies were excluded. Finally, 52 articles were eligible for this meta-analysis [4], [8], [9], [12]–[61] (Fig. 1), of which 11 were in English. All were published between 1996 and 2013 (Table 1).

**Table 1:** Main characteristics of the eligible studies.

Author(year)	No. of patients	Gender (male)		Age(year)		CBP time(min)		ACC time(min)		Ulinastatin (IU/kg)
Groups	Total	U	C	U	C	U	C	U	C	
Bao HJ (2006)	30	NA	NA	29.19±9.4	27.5±14.7	70.4±22.0	73.7±18.9	48.2±16.4	49.3±17.3	15000
Chen BJ (2011)	130	31	31	42.11±5.66	43.26±5.92	69.41±26.53	70.12±26.03	48.74±22.5	50.22±24.2	10000
Chen P (2003)	60	17	15	42±11	41±12	115±43	111±47	79±34	77±37	9×10^6^ IU/c
Chen RW (2007)	30	NA	NA	3.4±1.1	3.2±1.2	25.4±9.2	28.5±7.5	20.7±6.0	20.2±7.1	10000
Chen TT (2013)	60	14	12	49.8±10.8	50.4±10.0	102.5±20.7	99.5±22.6	74.7±20.5	69.0±20.4	12,000
Chen Y (2005)	24	6	9	10.25±5.53	9.58±7.09	46.26±6.58	47.37±17.22	25.08±6.44	27.26±10.16	10000
Gao XG (2012)	65	18	16	43±2	44±2	NA	NA	NA	NA	NA
Hiyama A (1997)	18	NA	NA	54±10	52±5	145±14	186±23	82±10	107±15	6×10^5^ IU/c
J BY (2007)	30	11	11	57.4±6.6	56.8±6.1	108±29	117±34	60±22	67±23	1×10^6^ IU/c
Ji HW (2009)	36	NA	NA	60±8	59±9	120±19	105±16	74±16	66±11	1×10^6^ IU/c
Jiang B (2012)	40	9	10	34±12	35±11	100±29	103±38	68±24	71±25	12000
Jiang CB (2009)	40	NA	NA	35.6±8.3	37.4±9.2	112.6±23.9	109.2±19.4	81.1±16.3	79.4±15.8	20000
Kawamura T (1996)	22	NA	NA	63±11	54±17	159±27	159±47.4	97±26	101±32.6	6000
Kuang X (2004)	40	NA	NA	30.19±19.4	28.5±14.7	70.4±22	74.7±18.9	49.2±16.4	50.5±17.3	15000
Li J (2010)	30	6	8	43±8	45±9	129±26	131±28	97±22	99±23	20000
Li JL (2009)	40	10	12	5.3±3.2	4.8±3.5	90.88±51.30	90.68±50.2	75.6±19.02	72±20.67	10000
Li LW (2004)	26	NA	NA	NA	NA	NA	NA	NA	NA	12000
Li MR (2005)	40	4	6	33.7±13.1	34.3±12.6	57.2±38.1	56.5±37.6	35.5±23.6	36.2±22.1	12000
Li Q (2012)	60	14	14	40.5±5.92	46.3±6.25	59.5±19.9	61.6±19.1	44.7±17.0	45.5±16.5	10000
Li WD (2009)	56	14	16	17.8±7.6	17.8±8.1	41.9±5.8	42.3±6.7	26.3±3.7	25.7±3.3	12000
Li YC (2004)	20	4	4	50.5±7.6	46.7±10.8	92.8±21	98.3±25.8	63.5±19.1	69.6±22.2	12000
Liu JD (2009)	36	8	9	51.8±10.6	50.4±10	95.9±22.4	98.6±20.9	69.2±21.7	67.9±19.4	12000
Liu Y (2005)	20	4	5	32.1±8.7	31.2±9.5	71.9±19.5	73.5±18.1	52±12.5	53.2±10.3	3×10^5^ IU/c
Meng DM (2002)	20	NA	NA	NA	NA	NA	NA	NA	NA	12000
Nakanishi K (2006)	28	12	11	62±9	61±10	150±37	135±39	118±32	104±32	5000
Oh SY (2012)	60	20	16	67±19	60±12	99±25	96±22	74±19	76±21	1×10^6^ IU/c
Park JB (2013)	110	10	27	55.37±14.22	47.55±13.86	125.85±37.58	116.91±32.40	78.13±27.64	70.83±27.00	5000
Qi XY (2010)	60	27	24	60.3±5.2	61.3±4.7	121.7±26.3	108.8±25.6	68.7±19.6	57.2±24.1	1×10^6^ IU/c
Qiang Z (2004)	30	7	6	12±5.4	11.8±5.2	87±28	82±27	51±20	46±21	20000
Ren BH (2004)	30	8	7	42.4±5.64	44.06±5.96	93.6±16.4	94.87±16.49	48.27±10.99	54.67±9.09	12000
Shao B (2006)	16	3	3	44.0±5.29	43.13±5.37	96.75±14.06	98.25±15.96	66.50±7.60	64.63±8.73	12000
Shu Q (2005)	27	7	8	3.63±1.81	4.45±4.04	46.43±7.06	45.67±8.86	22.14±3.66	24.45±8.08	15000
Song JE (2011)	48	8	9	54±16.3	52±17.7	170±29.6	172±26.6	102±22.2	105±24.4	5,000
Song JE (2013)	24	4	6	58±17	59±15	150±44	139±42	104±39	91±44	5,000
Sugita T (2002)	22	NA	NA	5.2±3.4	4.9±3.8	96.1±61.3	117.7±61.6	56.3±44.1	69.5±44.3	5000
Sun CY (2003)	20	5	6	19.7±5.6	16.3±4.4	59.9±7.6	71.9±7.2	46.2±7.2	49.8±8.6	8000
Sun CY (2003)-1	20	6	6	19.9±5.1	16.3±4.4	64.2±8.8	71.9±7.2	40.2±7.8	49.8±8.6	16000
Wang DJ (2005)	30	7	6	35.9±12.4	39.1±14.3	68.2±28.1	82.1±34.2	48.8±24.6	57.7±29.5	20000
Wang X (2012)	24	6	5	47.3±16.8	45.6±18.2	71.3±18.6	69.2±19.1	49.9±18.3	48.1±17.2	15000
Wang YQ(2008)	42	10	9	7.5±3.7	6.9±2.8	NA	NA	NA	NA	20000
Wei L(2009)	25	NA	NA	35.1±14.1	30.2±10.6	78.5±26.6	72.1±35.8	48.7±24.5	42.6±27.8	20000
Xu CE(2013)	36	15	16	54.8±8.9	53.2±7.3	235.5±25.9	247.2±20.3	87.3±23.7	89.4±24.6	20,000
Xu KQ (2004)	40	8	11	46.1±12.3	45.7±11.8	71.09±11.42	70.28±10.74	40.88±9.75	41.71±8.92	10000
Xu KQ (2004)-1	40	8	11	47.2±10.9	45.7±11.8	69.87±10.91	70.28±10.75	41.67±9.43	41.71±8.92	20000
Xue QH (2006)	30	6	7	48.8±7.57	43.2±8.38	96±35	86±22	68±28	61±19	6000
Yu XY (2009)	28	NA	NA	48.89±8.74	52.33±9.17	58.11±18.16	56.11±18.92	43.33±15.75	42.56±16.21	8000
Yu XY (2009)-1	28	NA	NA	53.22±9.74	52.33±9.17	55.78±17.72	56.11±18.92	42.22±17.44	42.56±16.21	12000
Zhang JW (2004)	24	NA	NA	NA	NA	NA	NA	NA	NA	5000
Zhang JW (2004)-1	24	NA	NA	NA	NA	NA	NA	NA	NA	10000
Zhang XL (2007)	30	7	6	43.8±12.5	42.2±11.8	83.4±16.5	84.1±15.7	54.5±12.7	52.7±14.5	20000
Zhao QF (2007)	30	8	9	30±11	29±11	57±16	54±18	35±12	35±16	10000
Zhao QF (2007)-1	30	7	9	29±10	29±11	58±16	54±18	36±12	35±16	20000
Zhao Y (2009)	40	8	11	32±7	32±8	98±8	98±5	56±6	61±7	20000
Zhong JY (2007)	30	10	9	42±9	51±11	118.4±28.3	117.7±27.7	79.7±34.6	77.6±29	20000
Zhou Q (2010)	40	NA	NA	NA	NA	NA	NA	NA	NA	15000
Zou DQ (2005)	24	6	7	35±5	36±6	89±11	87±12	56±7	52±8	24000

Abbreviations: U, Ulinastatin; C, Control; CPB, Cardiopulmonary bypass; ACC, aortic cross clamping; OR, odds ratio; MD, mean difference; IU/c, Unit per case; NA, not available.

A total of 2025 patients were included in our meta-analysis; 1013 received ulinastatin and 1012 received a placebo saline solution. The groups showed no significant differences with respect to gender [OR (95% CI) were 0.85 (0.69, 1.04), P = 0.12], age [MD (95% CI) were -0.14 (−0.58, 0.31), P = 0. 55], CPB time [MD (95% CI) were −0.95 (−2.34, 0.44), P = 0.18], or aortic cross-clamping time [MD (95% CI) were −0.48 (−1.52, 0.57), P = 0.37] (Table 2). As no heterogeneity existed among these studies, a fixed-effect model was adopted.

**Table 2:** Meta-analysis of interesting outcomes between ulinastatin and control group in patients with CPB.

Outcomes	Studies (n)	Ulinastatin (n)	Control (n)	Heterogeneity		Meta-analysis Model	OR*/MD (95%CI)	*P*
				I^2^	P			
Gender(male)	39	393	420	0%	1.00	M-H, Fixed	0.85 [0.69, 1.04]*	0.12
Age(year)	48	980	978	9%	0.29	IV, Fixed	−0.14 [−0.58, 0.31]	0.55
CBP time(min)	47	998	998	17%	0.15	IV, Fixed	−0.95 [−2.34, 0.44]	0.18
ACC time(min)	46	958	963	7%	0.34	IV, Fixed	−0.48 [−1.52, 0.57]	0.37
TNF-α	15	349	334	92%	<0.01	Random	−7.53 [−9.45, −5.61]	<0.01
IL-6	14	324	309	80%	<0.01	Random	−32.87 [−39.53, −26.20]	<0.01
IL-8	12	287	271	94%	<0.01	Random	−2.40 [−3.40, −1.41]	0.02
IL-10	4	55	55	86%	<0.01	Random	14.49 [4.57, 24.42]	0.02
LDH (IU/L)	4	87	78	42%	0.16	IV, Fixed	−63.54 [−79.36, −47.72]	<0.01
CK(IU/L)	4	63	63	0%	0.99	IV, Fixed	−224.99 [−304.83, −145.14]	<0.01
CK-MB(ng/ml)	12	274	291	91%	<0.01	IV, Random	−8.75 [−14.23, −3.28]	<0.01
TnI(ng/ml)	8	175	197	6%	0.39	IV, Fixed	−0.14 [−0.20, −0.09]	<0.01
HR(beats/min)	8	175	200	0%	0.65	IV, Fixed	−1.63 [−4.11, 0.84]	0.20
MAP(mmHg)	8	136	133	0%	0.58	IV, Fixed	2.50 [0.19, 4.80]	0.03
MPAP(mmHg)	2	43	41	0%	0.37	IV, Fixed	0.84 [−0.64, 2.32]	0.26
CVP(cmH_2_O)	2	43	41	0%	0.52	IV, Fixed	0.89 [−0.07, 1.84]	0.07
Cardiac Index(L/min/m^2^)	3	57	55	11%	0.32	IV, Fixed	−0.18 [−0.42, 0.06]	0.14
LVEF (%)	2	44	44	0%	0.47	IV, Fixed	1.32 [−2.63, 5.26]	0.51
Auto Resuscitation(n)	9	175	166	0%	0.85	M-H, Fixed	1.98 [1.19, 3.30]*	<0.01
Patients Inotrope (n)	5	132	123	0%	0.71	M-H, Fixed	0.98 [0.52, 1.85] *	0.96
Postoperative Bleeding(ml)	6	136	162	61%	0.03	IV, Random	14.98 [−69.10, 99.07]	0.73
Complications(n)	7	467	545	0%	0.50	M-H, Fixed	0.68 [0.44, 1.04]*	0.07
MVT(h)	12	305	292	94%	<0.01	IV, Random	−3.29 [−4.41, −2.17]	<0.01
ITT(h)	7	167	195	57%	0.03	IV, Random	−1.58 [−2.84, −0.32]	<0.01
ICU Stay(h)	13	276	303	68%	<0.01	IV, Random	−2.17 [−8.13, 3.80]	0.48
Hospital Stay(d)	3	55	55	0%	0.72	IV, Fixed	0.43 [−1.93, 2.79]	0.72

Abbreviations: CPB, cardiopulmonary bypass; ACC, aortic cross-clamping; LDH, lactate dehydrogenase; CK-MB, creatine kinase-MB; TnI, troponin I; HR, heart rate; MAP, mean arterial pressure; MPAP, mean pulmonary arterial pressure; CVP, central venous pressure; LVEF, left ventricular ejection fraction; MVT: mechanical ventilation time; ITT: intubation time; ICU, intensive care unit; OR*, Odds Ratio; M-H, Mantel-Haenszel; IV, Inverse Variance; MD, mean difference; CI, confidence interval.

### Risk of bias of included trials

**Figure 2:**
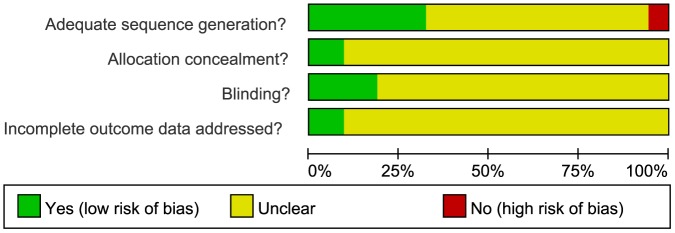
Methodological quality graph summarizing the risk of bias from all included studies.

Overall, 2 trials had a low risk of bias; 47 trials had a moderate risk of bias, due to insufficient information about the sequence generation process, concealed allocation methods, or blinding, and incomplete addressing of outcome data; and 3 trials [4], [26], [29] had a high risk of selection bias, as the patients' ID numbers, the order of admission, or specific groups of people were used for the generation of the randomization sequence (Fig. 2, S1 Figure).

### Efficacy of ulinastatin on patients with cardiopulmonary bypass

#### Inflammatory mediators (TNF-α, IL-6, IL-8 and IL-10)

At first, we perform a meta-analysis to assess the changes of these inflammatory mediators at twenty-four hours postoperatively. The heterogeneities were found, and the random-effect model was used to perform meta-analysis. Pooled analysis showed the significant differences existed in TNF-α (MD-7.53, 95%CI [−9.45, −5.61], P<0.01), IL-6 (MD-32.87, 95%CI [−39.53, −26.20], P<0.01), IL-8 (MD-2.40, 95%CI [−3.40, −1.41], P = 0.02) and IL-10 (MD14.49, 95%CI [4.57, 24.42], P = 0.02) between the ulinastatin and control groups (Table 2). Sensitivity analysis showed that the overall effect was not changed by omitting some studies until these heterogeneities became acceptable (heterogeneity: P>0.10; I^2^<50%) (Table 3).

**Table 3:** Sensitivity analyses of high heterogeneity outcomes in meta-analysis.

Heterogeneity outcomes	Omitted (excluded) studies	Ulinastatin (n)	Control (n)	Heterogeneity		Meta-analysis model	Outcomes	
				I^2^	*P*		MD (95%CI)	*P*
TNF-α(pg/ml)	7[15], [17], [18], [36], [48], [57], [59]	137	136	47%	0.06	IV, Fixed	−0.78 [−1.03, −0.52]	<0.01
IL-6(pg/ml)	5[18, 33, 36,44 53]	226	216	42%	0.08	IV, Fixed	−1.41 [−1.62, −1.20]	<0.01
IL-8(pg/ml)	4[17], [49], [51], [57]	169	163	42%	0.09	IV, Fixed	−0.69 [−0.92, −0.47]	<0.01
IL-10(pg/ml)	2[46], [55]	25	25	0%	0.61	IV, Fixed	2.39 [1.63, 3.14]	<0.01
CK-MB(ng/ml)	6[9], [12], [13], [14], [18], [27]	123	116	42%	0.11	IV, Fixed	−14.19 [−16.01, −12.37]	<0.01
MVT(h)	4[8], [17], [21], [22]	172	169	47%	0.06	IV, Fixed	−2.32 [−2.71, −1.93]	<0.01
ITT(h)	1[9]	126	126	40%	0.14	IV, Fixed	−2.43 [−2.93, −1.92]	<0.01
Postoperative Bleeding(ml)	1[21]	116	142	0%	0.79	IV, Fixed	56.79 [−2.09, 115.67]	0.06
ICU stay(h)	1[8]	258	285	0%	0.58	IV, Fixed	1.69 [−1.32, 4.70]	0.27

Abbreviations: TNF-α, tumor necrosis factor-α; IL, interleukine; CK-MB, creatine kinase-MB; ITT: intubation time; MVT, mechanical ventilation time; ICU, intensive care unit; IV, Inverse Variance; MD, mean difference; CI, confidence interval.

#### Myocardial Biomarkers (LDH, CK, CK-MB, and TnI)

We assessed these myocardial biomarkers: LDH changes in 4 trials, CK changes in 4 trials, CK-MB changes in 12 trials, and TnI changes in 8 trials. The meta-analysis demonstrated that all biomarkers significantly reduced in the ulinastatin group compared with the placebo groups. The MD (95% CI) values were −63.54 (−79.36, −47.72) for LDH (P<0.01), −224.99 (−304.83, −145.14) for CK (P<0.01), −8.75 (−14.23, −3.28) for CK-MB (P<0.01) and −0.14 (−0.20, −0.09) for TnI (P<0.01) (Table 2). As there was significant heterogeneity in the CK-MB values (P<0.01, I^2^ = 91%), a random-effect model was adopted. Sensitivity analysis showed that the overall effect was not changed by omitting 6 studies until these heterogeneities became acceptable (heterogeneity: P>0.10; I^2^<50%) (Table 3) [4].

#### Hemodynamic Parameters (HR, MAP, MPAP, and CVP)

Parameters relating to hemodynamics were measured in some trials after CPB. The meta-analysis results showed that ulinastatin had no significant effect on any of these parameters except MAP. The MD (95% CI) values were −1.63 (−4.11, 0.84) for HR (P = 0.20), 0.84 (−0.64, 2.32) for MPAP (P = 0.26), 0.89 (−0.07, 1.84) for CVP (P = 0.07), Although MAP showed a statistically significant increase—MD (95% CI) 2.50 (0.19, 4.80), P = 0.03—the clinical significance was unclear. Since there was no significant heterogeneity among these analyses, a fixed-effect model was adopted (Table 2).

#### Cardiac Function (Cardiac index and LVEF)

Only 3 trials reported the cardiac index and 2 trials the LVEF postoperatively. The meta-analysis showed no significant effects on either parameter: MD (95% CI) were −0.18 (−0.42, 0.06) for cardiac index (P = 0.14), and 1.32 (−2.63, 5.26) for left ventricular ejection fraction (P = 0.51). As there was no significant heterogeneity among these analyses, a fixed-effect model was adopted (Table 2).

#### Important Clinical Outcomes (Including the number of heart auto resuscitations, the number of patients needing inotropes, the postoperative bleeding volume, and the number of complications)

**Figure 3:**
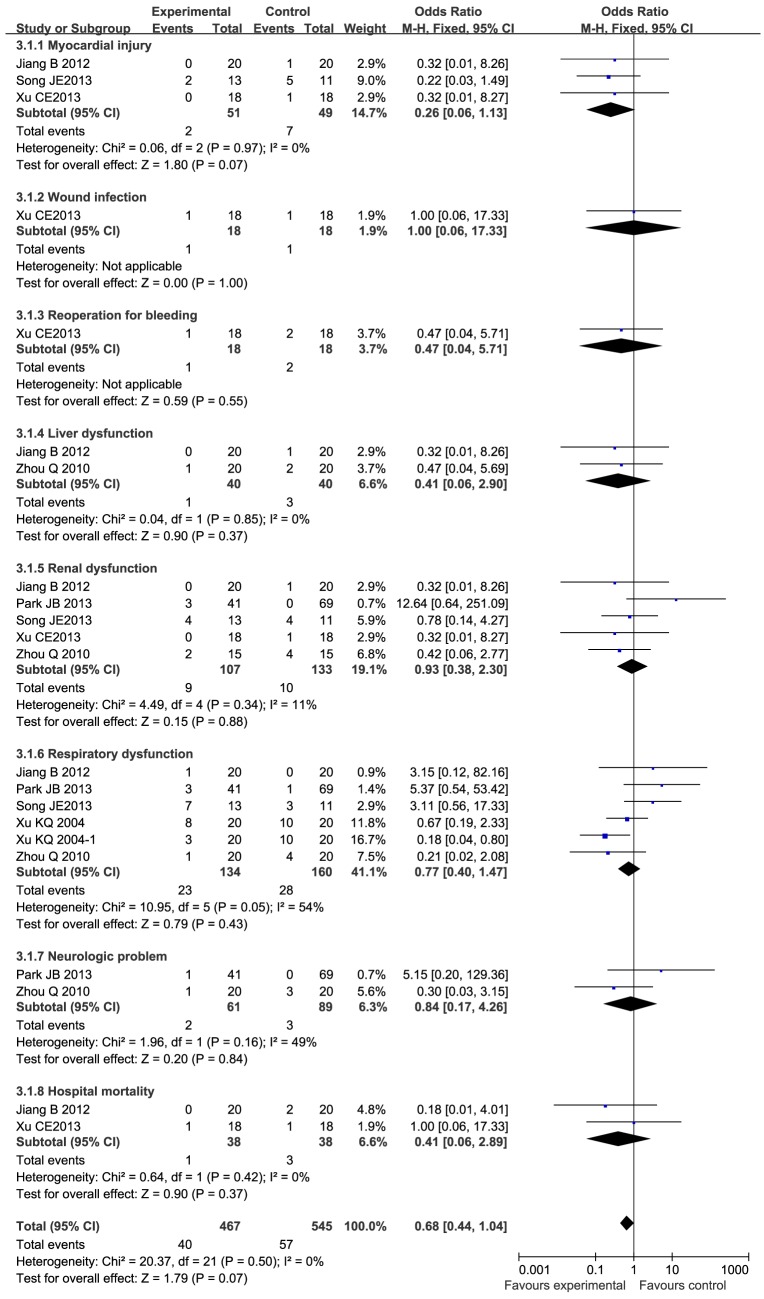
Forest plot of complications in patients who received ulinastatin versus controls in open-heart surgery with CPB. OR, Odds Ratio; M-H, Mantel-Haenszel; MD, mean difference; CI, confidence interval.

These important clinical outcomes have aroused the concern of clinicians. The results showed that the frequency of autoresuscitation increased significantly (OR 1.98, 95%CI 1.19 to 3.30, P<0.01) in the ulinastatin group, but there were no statistically significant differences in the number of patients needing inotropes (OR: 0.98, 95%CI 0.52 to1.85, P = 0.96), the postoperative bleeding (MD 14.98, 95%CI −69.10 to 99.07, P = 0.73), or the postoperative complications (MD 0.68, 95%CI 0.44 to 1.04, P = 0.07) (Table 2). Postoperative complications, including the number of cases with myocardial injury, wound infection, reoperation for bleeding, liver dysfunction, renal dysfunction, respiratory dysfunction, and neurological problems, as well as the in-hospital mortality, are shown in detail in Fig. 3. Heterogeneity was seen only in the postoperative bleeding (P = 0.03, I^2^ = 61%), where a random-effect model was adopted. Sensitivity analysis showed that the overall effect was not changed by omitting one study (MD 56.79, 95%CI −2.09 to 115.67, P = 0.06) (Table 3).

#### Duration of Mechanical Ventilation and Intubation

Eleven trials reported mechanical ventilation time (MVT), and 7 trials reported intubation time (ITT). The meta-analysis results showed that the durations of mechanical ventilation and intubation were significantly shortened in the ulinastatin group compared with the placebo group; MD (95% CI) values were −3.29 (−4.41, −2.17) and −1.58 (−2.84, −0.32) respectively (P<0.01) (Table 2). Significant heterogeneities were found (heterogeneity: P = 0.03, I^2^ = 57% in the duration of intubation and P<0.01, I^2^ = 94% in the duration of mechanical ventilation), and a random-effect model was adopted. Sensitivity analysis showed that the overall effects on the durations of intubation and mechanical ventilation were not altered significantly (still P<0.01) (Table 3).

#### Length of ICU and Hospital Stays

Finally, we assessed the effect of ulinastatin on the length of the postoperative ICU and hospital stays. Thirteen studies reported the length of stay in the ICU, and 3 studies reported the length of stay in the hospital. The meta-analysis indicated that there were no significant differences in the duration of either ICU stay (P = 0.48) or hospital stay (P = 0.72) between the ulinastatin and placebo groups: the MD (95% CI) values were −2.17 (−8.13, 3.80) and 0.43 (−1.93, 2.79), respectively. Significant heterogeneity was found in the ICU stay analysis (heterogeneity: P<0.01, I^2^ = 68%), and a random-effect model was adopted (Table 2). After one obviously different study was omitted, the heterogeneity became non-significant (heterogeneity: P = 0.58, I^2^ = 0%), and the same result was still obtained (P = 0.27) (Table 3).

### Publication bias

We assessed the publication biases in the analysis of the above 10 studies, Publication biases for two interesting outcomes (TnI, MVT) were detected by Begg's test or Egger's test (P<0.05).

## Discussion

Many studies have claimed that the postoperative complications of cardiac surgery are associated with the activation of inflammatory mediators and the occurrence of systemic inflammatory response syndrome (SIRS). Protease inhibitors have been used clinically to treat many diseases, such as HCV [62], HIV [63], and H1N1 virus [64], and also have a potential role as anti-tumor agents [65], [66] and parasiticides [67]. Ulinastatin is a multivalent serine protease inhibitor. It is a glycosylated protein composed of 143 amino acid residues and weighted from 62 kDa to 72 kDa, injected to treat various inflammatory diseases [68]. In CPB, it has been widely used to block the inflammatory cascade with a view to prevent postoperative complications. Several animal studies found that mediators of inflammation after CPB reduced significantly with ulinastatin treatment [69]. The same findings were demonstrated in human studies [70], [71].

As inflammatory mediators and proinflammatory cytokines, TNF-α, IL-6, IL-8, and PMNE can lead to an inflammatory cascade by acting on other inflammatory cytokines or influencing the actions of each other. In this study, we found that in patients undergoing cardiac surgery with CPB, the preoperative administration of ulinastatin significantly reduced the levels of these inflammatory mediators, as compare with those in the control group, although not to the baseline preoperative levels at twenty-four hours postoperatively. Moreover, IL-10, also known as the human cytokine synthesis inhibitory factor, is an anti-inflammatory cytokine that is capable of inhibiting the synthesis of proinflammatory cytokines such as IFN-γ, IL-2, IL-3, and TNF-α. The levels of IL-10 increase in response to increased levels of proinflammatory cytokines [72]. In our study, the levels of IL-10 also increased after CPB; however, this increase was greater in the ulinastatin groups than in the control groups. Therefore, we consider that the anti-inflammatory function of ulinastatin occurs by blocking the inflammatory cascade rather than by clearing of inflammatory mediators.

In general, the key clinical and prognostic parameters, such as hemodynamics, organ function, duration of mechanical ventilation, and length of ICU or hospital stays, have received more attention from surgeons. Some studies showed that ulinastatin can reduce the risk of myocardial injury in an animal model [73], [74] and in patients undergoing CPB [5], but other studies found that this effect was not remarkable [9], [12], [14]. In this meta-analysis, we focused on biomarkers of myocardial damage and some important clinical outcomes. The results showed a significant decrease in these biomarkers (including LDH, CK, CK-MB, and TnI) in patients treated with ulinastatin. However, we did not find a significant improvement in cardiac function (cardiac index and LVEF) or hemodynamic parameters (MPAP, CVP). Although there was a statistically significant increase in MAP, the clinical significance was not certain, because the magnitude of the increase in the MD value was only 2.5 mmHg. This meta-analysis also showed that ulinastatin may increase the frequency of heart auto resuscitation, and shorten the duration of mechanical ventilation and intubation, but does not change the number of patients needing inotropic agents, the volume of postoperative bleeding, or the complication rate, and does not shorten the ICU or hospital stay.

Cardiac insufficiency, such as postoperative low cardiac output or even cardiogenic shock, is the most common complication after cardiac surgery with CPB. Regional myocardial ischemia/reperfusion injury in the process of aortic cross clamping and opening, together with the apoptosis or necrosis of cardiomyocytes, are thought to be critical reasons for cardiac insufficiency [75], [76]. Additionally, the activation of inflammatory mediators, finally leading to SIRS, is also thought to be an important factor [76]. These biomarkers, especially CK-MB and cTnI, are predictive factors that reflect myocardial injury [19]; cTnI is the most sensitive indicator of minor myocardial damage and has superior cardiac specificity to CK-MB [77]. Therefore, the protective effect of ulinastatin on myocardium may be related with inhibition or elimination of the inflammatory mediators and alleviation of SIRS. However, cardiac function was not obviously improved in the ulinastatin group, while hemodynamic parameters, which are affected by many factors closely related with cardiac function, blood volume, and systemic inflammatory reaction, also did not improve significantly. Another interesting finding was that ulinastatin did not reduce the number of patients who needed inotropic agents, again suggesting that it did not ameliorate cardiac function, although it may have a benefit in limiting myocardial damage.

Successful auto resuscitation can avoid electrical defibrillation, which may cause damage to the myocardium. Many factors, such as the cardiac conduction system, blood electrolytes, and acid-base balance, are related with the heart's auto resuscitation rate [78]. Nine trials reported data concerning heart auto resuscitation intraoperatively. Overall, the auto resuscitation rate in the ulinastatin group was 80% (140/175), significantly higher than in the control group 66.87% (111/166): OR = 1.98, 95%CI (1.19, 3.30), P<0.05. Ulinastatin's beneficial effect on the heart's autoresuscitation rate may be associated with its alleviation of damage in cardiomyocytes, including the cells of the cardiac conduction system.

We found no significant difference in complications (including key organ function, postoperative bleeding, wound infection and hospital mortality) between the 2 groups according to the current data. However, very few studies in this meta-analysis reported these data; thus, more studies and a larger sample size will be needed before we can discuss the relationship between ulinastatin and complications.

In our study, the outcomes of interest were improved by ulinastatin compared with placebo control as regards the duration of mechanical ventilation and intubation, whereas no effect was noted on the length of ICU or hospital stays. According to a sensitivity analysis, the outcomes were not changed. Some studies found that ulinastatin can reduce pulmonary injury and improve pulmonary function [8], [42], perhaps via a reduction in inflammatory mediators. However, these clinical outcomes are influenced by many factors and a prospective, multicenter, randomized controlled trial is mandated to elucidate these matters.

The heterogeneity of some subgroup analyses was significant. Possible reasons are that the ulinastatin dosage may affect the result, and different dosages were used to investigate the effects. The doses of ulinastatin ranged from 5000 U/kg to 20,000 U/kg, with total doses reported to range from 600,000 U to 1,000,000 U. It seems that a higher dosage produced better effects [8], but no studies discussed the dose-effect relationship. In addition, different drug delivery protocols were used. In some studies, the ulinastatin was administered 3 times (preoperative, operative, and postoperative) [13]; in other studies, it was given twice (preoperatively and postoperatively) [42], or delivered only once by being added to the CPB pump prime [9] or via continuous intravenous infusion [79]. Some researchers think that ulinastatin preprocessing (used preoperatively) can achieve more advantages [80]. In order to explain the effect of ulinastatin adequately, a subgroup analysis is required, taking into account the methods of administration or dosage, but this was not possible in the present study because of the limited quantity of data.

Our study had some limitations and potential biases. First, in our meta-analysis, only randomized controlled trials in English and Chinese were included. However, ulinastatin was allowed first in Japan and is now widely used in Asia. As many studies have been published in the Japanese or Korean languages, there may have been bias owing to the language restriction. Second, in some of these randomized controlled trials, the aspects of the study design relating to patient inclusion, sequence generation, allocation concealment, and blinding were not reported clearly. In some trials, although the designs were reported, the methods adopted were different. Therefore, the quality of these trials was uneven, and this unevenness may have been a main source of heterogeneity. Third, data relating to the functional parameters of main organs were not reported adequately, so we were not able to analyze the effects of ulinastatin treatment on organ function after CPB. Fourth, the data regarding outcomes of interest did not include the same variables in every study, and some data needed to be estimated; thus, the accuracy of some results was affected to some extent. Last, the numbers of patients and studies included were insufficient, especially for subgroup analysis, so a further meta-analysis without language restrictions should be performed.

### Summary of findings

The proinflammatory cytokines (TNF-α, IL-6 and IL-8) were significantly reduced and the anti-inflammatory cytokine IL-10 were significantly increased at 24th hour of plasma levels in the ulinastatin-treated patients from China and Japan undergoing open-heart surgery with CPB.

Ulinastatin treatment had no significant effect on hemodynamics, cardiac function, patients' need for inotropes, complications, or postoperative course (ICU and hospital stay).

Ulinastatin treatment may have protective effects on myocardial injury, and can increase the frequency of auto resuscitation, shorten the duration of intubation and mechanical ventilation.

## Supporting Information

S1 Figure **Risk of bias summary: review authors' judgments about each risk of bias item for each included study.**
(EPS)

S1 PRISMA Checklist (DOC)

## References

[1] Butler J, Rocker GM, Westaby S (1993) Inflammatory response to cardiopulmonary bypass. The Annals of thoracic surgery 55:552–559. 8431082 10.1016/0003-4975(93)91048-r

[2] Laffey JG, Boylan JF, Cheng DC (2002) The systemic inflammatory response to cardiac surgery: implications for the anesthesiologist. Anesthesiology 97:215–252. 12131125 10.1097/00000542-200207000-00030

[3] Sablotzki A, Friedrich I, Muhling J, Dehne MG, Spillner J, et al (2002) The systemic inflammatory response syndrome following cardiac surgery: different expression of proinflammatory cytokines, procalcitonin in patients with and without multiorgan dysfunctions. Perfusion 17:103–109. 11958300 10.1177/026765910201700206

[4] Song J, Park J, Kim JY, Kim JD, Kang WS, et al (2013) Effect of ulinastatin on perioperative organ function and systemic inflammatory reaction during cardiac surgery: a randomized double-blinded study. Korean J Anesthesiol 64:334–340. 23646243 10.4097/kjae.2013.64.4.334 PMC3640166

[5] Wang GY, Qiu HB, Zhan SG, Li LH (2007) [Protection of ulinastatin against myocardial injury induced by off-pump coronary artery bypass graft surgery: report of 24 cases]. Zhonghua yi xue za zhi 87:2502–2504. 18067816

[6] Chen H, He MY, Li YM (2009) Treatment of patients with severe sepsis using ulinastatin and thymosin alpha1: a prospective, randomized, controlled pilot study. Chinese medical journal 122:883–888. 19493408

[7] Kanai T, Ishiwata T, Kobayashi T, Sato H, Takizawa M, et al (2011) Ulinastatin, a urinary trypsin inhibitor, for the initial treatment of patients with Kawasaki disease: a retrospective study. Circulation 124:2822–2828. 22104548 10.1161/CIRCULATIONAHA.111.028423

[8] Xu CE, Zou CW, Zhang MY, Guo L (2013) Effects of high-dose ulinastatin on inflammatory response and pulmonary function in patients with type-A aortic dissection after cardiopulmonary bypass under deep hypothermic circulatory arrest. Journal of cardiothoracic and vascular anesthesia 27:479–484. 23545347 10.1053/j.jvca.2012.11.001

[9] Park JB, Kim SH, Lee SA, Chung JW, Kim JS, et al (2013) Effects of ulinastatin on postoperative blood loss and hemostasis in atrioventricular valve surgery with cardiopulmonary bypass. The Korean journal of thoracic and cardiovascular surgery 46:185–191. 23772405 10.5090/kjtcs.2013.46.3.185 PMC3680603

[10] Hozo SP, Djulbegovic B, Hozo I (2005) Estimating the mean and variance from the median, range, and the size of a sample. BMC medical research methodology 5:13. 15840177 10.1186/1471-2288-5-13 PMC1097734

[11] Higgins JP, Altman DG, Gotzsche PC, Juni P, Moher D, et al (2011) The Cochrane Collaboration's tool for assessing risk of bias in randomised trials. Bmj 343:d5928. 22008217 10.1136/bmj.d5928 PMC3196245

[12] Song JE, Kang WS, Kim DK, Yoon TG, Kim TY, et al (2011) The effect of ulinastatin on postoperative blood loss in patients undergoing open heart surgery with cardiopulmonary bypass. The Journal of international medical research 39:1201–1210. 21986122 10.1177/147323001103900408

[13] Oh SY, Kim JC, Choi YS, Lee WK, Lee YK, et al (2012) Effects of ulinastatin treatment on myocardial and renal injury in patients undergoing aortic valve replacement with cardiopulmonary bypass. Korean journal of anesthesiology 62:148–153. 22379570 10.4097/kjae.2012.62.2.148 PMC3284737

[14] Nakanishi K, Takeda S, Sakamoto A, Kitamura A (2006) Effects of ulinastatin treatment on the cardiopulmonary bypass-induced hemodynamic instability and pulmonary dysfunction. Critical care medicine 34:1351–1357. 16540949 10.1097/01.CCM.0000215110.55899.AE

[15] Chen TT, Jiandong L, Wang G, Jiang SL, Li LB, et al (2013) Combined treatment of ulinastatin and tranexamic acid provides beneficial effects by inhibiting inflammatory and fibrinolytic response in patients undergoing heart valve replacement surgery. The heart surgery forum 16:E38–47. 23439357 10.1532/HSF98.20121072

[16] Bingyang J, Jinping L, Mingzheng L, Guyan W, Zhengyi F (2007) Effects of urinary protease inhibitor on inflammatory response during on-pump coronary revascularisation. Effect of ulinastatin on inflammatory response. The Journal of cardiovascular surgery 48:497–503. 17653011

[17] Chen BJ, Sun M, Shao J, Wang DJ (2011) Direct perfusion with hypothermic oxygenated blood mixed with ulinastatin through pulmonary artery to protect pulmonary function during cardiopulmonary bypass. The Journal of Practical Medicine 27:1765–1767.

[18] Chen P, Zhang XH, Chen WD, Luo ZC, Zhou CB, et al (2003) Effects of ulinastatin on myocardial protection in patients undergoing cardiac operation with cardiopulmonary bypass. Guangdong Medical Journal 24:281–283.

[19] Gao XG (2012) Protective Effect of Ulinastatin on Myocardium in Cardiopulmonary Bypass Surgery. Guide of China Medicine 10:75–76.

[20] Ji HW, Chen L (2009) [Effects of ulinastatin on coagulation and platelet function in patients undergoing coronary artery bypass grafting with cardiopulmonary bypass]. Zhonghua yi xue za zhi 89:175–178. 19537033

[21] Jiang B, Jin ZX, Yi W, Ren C, Bai L, et al (2012) The myocardial and blood protective effects of ulinastatin combined with tranexamic acid in patients underwent valve replacement surgery with cardiopulmonary bypass. Chinese Journal of Extracorporeal Circulation 10:207–211.

[22] Li J, Pan JH, Kang F, Chen KZ (2010) Effects of penehyclidine hydrochloride combined with ulinastatin on lung injury in patients undergoing cardiac valve replacement with cardiopulmonary bypass. Chinese Journal of Anesthesiology 30:1420–1423.

[23] Li LW, Li L, Shen JM, He XJ, Zhou JM (2004) The Protective Effect of Ulinastatin on Myocardial Reperfusion Injury during Cardiopulmonary Bypass in Patients Underwent Valve Replacement. Journal of Chinese Physician 6:1642–1643.

[24] Li Q, Liu Y, Liu S, Yu D, Chen ZY (2012) The protective effect of ulinastatin on lung and kidney for patients with mitral valve replacement. Chinese Journal of Experimental Surgery 29:1184–1186.

[25] Meng DM, Yu JG, Zhou GL, Suai XJ, Lei WF, et al (2002) Myocardial protective effect of ulinastatin during open heart surgery with cardiopulmonary bypass. Chinese Journal of Anesthesiology 22:344–347.

[26] Qi XY, Hu QF, Huang WQ (2010) Myocardial Protection of Ulinastatin in Patients undergoing Coronary Artery bypass Grafting with Cardiopulmonary Bypass. Herald of Medicine 29:477–479.

[27] Sun CY (2003) Effects of ulinastatin with different doses on myocardial protection for cardiac surgery with cardiopulmonary bypass. Anesthesiology. Jilan: Sandong University. pp.38.

[28] Wang X, Yin LJ, Zhang SL (2012) Myocardial Protection of Ulinastatin in Cardiopulmonary Bypass in Patients. The Journal of Medical Theory and Practice 25:1808–1809.

[29] Wang YQ, Chen YX (2008) Protective effect of ulinastatin on lung injury induced by cardiopulmonary bypass. Shaanxi Medical Journal 37:1620–1622.

[30] Xu KQ, Shun PW, Huang WQ, Chen BX, Tan JF, et al (2004) Effects of ulinastatin on lung function after cardiopulmonary bypass. Chinese Journal of Thoracic and Cardiovascular Surgery 20:7–9.

[31] Zhang JW, Chen Y, Zhu M, Gu HB (2004) Lung protection of different dosages of ulinastatin in infant heart operation under cardiopulmonary bypass. Academic Journal of Shanghai Second Medical University 22:933–935.

[32] Zhou Q, Wang G, Gao CQ, Chen TT (2010) Effect of ulinastatin on perioperative inflammatory response to coronary artery bypass grafting with cardiopulmonary bypass. Journal of Central South University(Medical Science) 35:107–110. 10.3969/j.issn.1672-7347.2010.02.003 20197607

[33] Jiang YF, Wang WW, Ye Wl, Ni YF, Li J, et al (2008) Effects of alprostadil and ulinastatin on Inflammatory response and lung injury after cardiopulmonary bypass in pediatric patients with congenital heart diseases. National Medical Journal of Chian 88:2893–2897. 19080093

[34] Jiang CB, Cheng DZ, He ZF, Liu Y, Jiang XF, et al (2009) Effect of ulinastatin on plasma tumor necrosis factor-alpha and A-aDO2 in patients undergoing mitral valve and aortic valve replacement under cardiopulmonary bypass. The Journal of Practical Medicine 25:1860–1862.

[35] Ren BH, Jing H, Li DM, Li ZD (2004) The protective effect of ulinastatin on pulmonary function during cardiopulmonary bypass. Journal of Medical Postgraduates 17:519–524.

[36] Li WD (2009) Effect of Ulinastatin on Inflammatory Response and Renal Function in Pediatric Cardiopulmonary Bypass Surgery. Journal of Clinical Research 26:1014–1016.

[37] Chen RW, Gao JP, Liu PB, Zhou WW, Wang JH (2007) The combined effects of amphylline and ulinastatin to inflammatory cytokines in infants undergoing cardiac operation under cardiopulmonary bypass. Journal of Clinical Pediatric Surgery 6.

[38] Li YC, Jiang Z, Jin XH, Luo J, Guo KF (2004) Protective effect of ulinustatin on pulmonary function during cardiopulmonary bypass. Journal of Clinical Anesthesiology 20:69–72.

[39] Chen Y, Zhang JW, Zhou H, Bai J (2005) Application of ulinastatin during open-heart surgery under cardiopulmonary bypass in infants. Journal of Clinical Anesthesiology 21:527–530.

[40] Zou DQ, Zhou JM, Chang YT, He XJ, Yuan GX, et al (2005) Effects of ulinastatin on cerebral inflammatory response during cardiopulmonary bypass. Journal of Central South University(Medical Science) 30:420–423. 16190388

[41] Wang DJ, Liu JX, Yin BL (2005) Protective effects of ulinastatin on lung injury during cardiopulmonary bypass. Journal of Central South University(Medical Science) 30:670–672. 16708806

[42] Sugita T, Watarida S, Katsuyama K, Nakajima Y, Yamamoto R, et al (2002) Effect of a human urinary protease inhibitor (Ulinastatin) on respiratory function in pediatric patients undergoing cardiopulmonary bypass. The Journal of cardiovascular surgery 43:437–440. 12124548

[43] Hiyama A, Takeda J, Kotake Y, Morisaki H, Fukushima K (1997) A human urinary protease inhibitor (ulinastatin) inhibits neutrophil extracellular release of elastase during cardiopulmonary bypass. Journal of cardiothoracic and vascular anesthesia 11:580–584. 9263089 10.1016/s1053-0770(97)90008-2

[44] Zhong JY, Li JL, Yang CX (2007) Effects of ulinastatin on renal function cardiopulmonary bypass in patients undergoing valve replacement. International Journal of Anesthesiology and Resuscitation 28:32–34.

[45] Li MR, BL L, Zhang XY, Zhan H (2005) Effects of ulinastatin on systemic inflammatory response during cardiopulmonary bypass. Chinese Journal of Primary Medicine and Pharmacy 12:1157–1158.

[46] Qiang Z, Zhou PJ, Li YL, Li TC, Chen CH (2004) Effects of ulinastatin on inflammatory factors during cardiopulmonary bypass. Chinese Journal of Postgraduates of Medicine 27:24–28.

[47] Wei L, Liu B, Liang YN, Chen YJ, Chen H, et al (2009) The Protective Effects of Ulinastatin on Pulmonary Tissue during Cardiopulmonary Bypass. Chinese Journal of Extracorporeal Circulation 7:4–7.

[48] Liu JD, Wang G, Chen TT, Zhou Q, Gao CQ, et al (2009) Effects of Ulinastatin on Inflammatory Response to Cardiopulmonary Bypass in Cardiac Valve Replacement Surgery. Chinese Journal of Extracorporeal Circulation 7:12–15.

[49] Li JL, Zhao YL, Li XL, Zhao ZY, Liu XM, et al (2009) Effects of Ulinastatin on NF -κB Activity of Polymorphonuclear Neutrophils, TNF-a and IL-8 in Patients Undergoing Cardiopulmonary Bypass. Chinese Journal of Extracorporeal Circulation 7:8–11.

[50] Zhang XL, Wang Y, Zhou HY, YAO YY, Ye HZ, et al (2007) Effect of ulinastatin on expression of S100β protein and TNF-α in patients undergoing open-heart surgery with cardiopulmonary bypass. Chinese Journal of Emergency Medicine 16:50–53.

[51] Shu Q, Yang XW, Shi Z, Shi SS, Zhang XH, et al (2005) Protective effects of ulinastatin on lung injury in pediatric patients undergoing cardiopulmonary bypass. Chinese Journal of Emergency Medicine 14:1027–1030.

[52] Bao HJ, Gao BS, Li TC, Zhu B, Liu DB, et al (2006) Protective Effects of Ulinastatin on Liver in Cardiopuimonary Bypass during Open Heart Surgery. Chinese Journal of Clinical Medicine 13:320–321.

[53] Shao B, Hu LD, Guo X, Liu QX, Xia X, et al (2006) Effects of ulinastatin on proinfiammatory cytokines in mitral valve replacement patients under hypothermic cardiopulmonary bypass. Chinese Journal of Cardiovascular Research 4:120–122.

[54] Zhao Y, Peng J, Yu HL, Kong H, Shi LJ, et al (2009) Effects of ulinastattn on systemic inflammatory response in patients undergoing cardiac valve replacement with cardiopulmonary bypass. Chinese Journal of Anesthesiology 29:685–687.

[55] Zhao QF, Hu XT, Du J, Wu GW (2007) Effects of ulinastatin and aprotinin on inflammatory response and myocardial injury in children undergoing cardiopulmonary bypass. Chinese Journal of Anesthesiology 27:199–203.

[56] Xu KQ, Chen BX, Huang WQ, Xia HJ, Hei ZQ, et al (2002) Effects of ulinastatln On lung inflammatory response to cardiopulmonary bypass. Chinese Journal of Anesthesiology 22:325–328.

[57] Yu XY, Fan LL (2009) Effects of different doses of ulinastatin on inflammatory response and pulmonary function after cardiopulmonary bypass. Chinese Critical Care Medicine 21:664–666. 19930883

[58] Xue QH, Chang KQ, Cheng WP (2006) Effects of Ulinastatin on Inflammatory Cytokins Levels in Bronchoalveolar Lavage Fluid and Pulmonary Function in Patients Undergoing Cardiopulmonary Bypass. Chinese Circulation Journal 21:297–300.

[59] Kuang X, Huang Y (2004) Influence of ulinastatin of the release of concentration of TNF-a, IL-6 and IL-8 levels in plasma after cardiopulmonary bypass. China Medical Engineering 12:26–28.

[60] Liu Y, Chen JH, Li YQ, Guo QL (2005) Effect of ulinastatin on cerebral inflammatory response in patients undergoing cardiopulmonary bypass. China Journal of Modern Medicine 15:1389–1391.

[61] Kawamura T, Inada K, Akasaka N, Wakusawa R (1996) Ulinastatin reduces elevation of cytokines and soluble adhesion molecules during cardiac surgery. Canadian journal of anaesthesia = Journal canadien d'anesthesie 43:456–460. 10.1007/BF03018106 8723851

[62] Talwani R, Heil EL, Gilliam BL, Temesgen Z (2013) Simeprevir: a macrocyclic HCV protease inhibitor. Drugs of today 49:769–779. 24524095 10.1358/dot.2013.49.12.2067249

[63] Ucciferri C, Falasca K, Vignale F, Di Nicola M, Pizzigallo E, et al (2013) Improved metabolic profile after switch to darunavir/ritonavir in HIV positive patients previously on protease inhibitor therapy. Journal of medical virology 85:755–759. 23508901 10.1002/jmv.23543

[64] Zhirnov OP, Matrosovich TY, Matrosovich MN, Klenk HD (2011) Aprotinin, a protease inhibitor, suppresses proteolytic activation of pandemic H1N1v influenza virus. Antiviral chemistry & chemotherapy 21:169–174. 21602614 10.3851/IMP1715

[65] Morjen M, Kallech-Ziri O, Bazaa A, Othman H, Mabrouk K, et al (2013) PIVL, a new serine protease inhibitor from Macrovipera lebetina transmediterranea venom, impairs motility of human glioblastoma cells. Matrix biology: journal of the International Society for Matrix Biology 32:52–62. 23262217 10.1016/j.matbio.2012.11.015

[66] Sierko E, Wojtukiewicz MZ, Zimnoch L, Ostrowska-Cichocka K, Tokajuk P, et al (2012) Protein Z/protein Z-dependent protease inhibitor system in human non-small-cell lung cancer tissue. Thrombosis research 129:e92–96. 21975032 10.1016/j.thromres.2011.09.005

[67] de Almeida Nogueira NP, Morgado-Diaz JA, Menna-Barreto RF, Paes MC, da Silva-Lopez RE (2013) Effects of a marine serine protease inhibitor on viability and morphology of Trypanosoma cruzi, the agent of Chagas disease. Acta tropica 128:27–35. 23770204 10.1016/j.actatropica.2013.05.013

[68] Matsuno YK, Nakamura H, Kakehi K (2006) Comparative studies on the analysis of urinary trypsin inhibitor (ulinastatin) preparations. Electrophoresis 27:2486–2494. 16786482 10.1002/elps.200500854

[69] Chen X, Wang Y, Luo H, Luo Z, Liu L, et al (2013) Ulinastatin reduces urinary sepsisrelated inflammation by upregulating IL10 and downregulating TNFalpha levels. Molecular medicine reports 8:29–34. 23685622 10.3892/mmr.2013.1480

[70] Hu CL, Xia JM, Cai J, Li X, Liao XX, et al (2013) Ulinastatin attenuates oxidation, inflammation and neural apoptosis in the cerebral cortex of adult rats with ventricular fibrillation after cardiopulmonary resuscitation. Clinics 68:1231–1238. 24141840 10.6061/clinics/2013(09)10 PMC3782733

[71] Li M, Yong-Zhe L, Ya-Qun M, Sheng-Suo Z, Li-Tao Z, et al (2013) Ulinastatin alleviates neuroinflammation but fails to improve cognitive function in aged rats following partial hepatectomy. Neurochemical research 38:1070–1077. 23508312 10.1007/s11064-013-1018-z

[72] Daryapeyma A, Aarstad HJ, Wahlgren CM, Jonung T (2014) Perioperative cytokine response to infection associated with elective arterial surgery. Vascular and endovascular surgery 48:116–122. 24270687 10.1177/1538574413512376

[73] Hu CL, Li H, Xia JM, Li X, Zeng X, et al (2013) Ulinastatin improved cardiac dysfunction after cardiac arrest in New Zealand rabbits. The American journal of emergency medicine 31:768–774. 23602756 10.1016/j.ajem.2012.11.012

[74] Chen SQ, Zhou SF, Li ZP, Zhang J, Yan P, et al (2008) [Protective effect of ulinastatin on myocardium after cardiopulmonary resuscitation in rats]. Zhongguo wei zhong bing ji jiu yi xue = Chinese critical care medicine = Zhongguo weizhongbing jijiuyixue 20:730–732. 19111120

[75] Emrich F, Walther T, Muth P, Ullmann C, Rastan AJ, et al (2012) Selective cerebral perfusion using moderate flow in complex cardiac surgery provides sufficient neuroprotection. Are children young adults? European journal of cardio-thoracic surgery: official journal of the European Association for Cardio-thoracic Surgery 42:704–710. 22843513 10.1093/ejcts/ezs119

[76] Meldrum DR, Donnahoo KK (1999) Role of TNF in mediating renal insufficiency following cardiac surgery: evidence of a postbypass cardiorenal syndrome. The Journal of surgical research 85:185–199. 10423318 10.1006/jsre.1999.5660

[77] He QL, Zhong F, Ye F, Wei M, Liu WF, et al (2014) Does intraoperative ulinastatin improve postoperative clinical outcomes in patients undergoing cardiac surgery: a meta-analysis of randomized controlled trials. BioMed research international 2014:630835. 24734237 10.1155/2014/630835 PMC3964764

[78] Sirlak M, Eryilmaz S, Yazicioglu L, Kiziltepe U, Inan MB, et al (2003) Conduction disturbances in coronary artery bypass surgery. International journal of cardiology 92:43–48. 14602215 10.1016/s0167-5273(03)00038-x

[79] Itaba S, Nakamura K, Aso A, Tokunaga S, Akiho H, et al (2013) Prospective, randomized, double-blind, placebo-controlled trial of ulinastatin for prevention of hyperenzymemia after double balloon endoscopy via the antegrade approach. Digestive endoscopy: official journal of the Japan Gastroenterological Endoscopy Society 25:421–427. 23368820 10.1111/den.12014

[80] Zhou Q, Wang G, Gao C, Chen T (2010) [Effect of ulinastatin on perioperative inflammatory response to coronary artery bypass grafting with cardiopulmonary bypass]. Zhong nan da xue xue bao Yi xue ban = Journal of Central South University Medical sciences 35:107–110. 20197607 10.3969/j.issn.1672-7347.2010.02.003

